# Self‐Leadership and Caring as Foundations of Relational Accountability in Nursing Clinical Judgement: A Conceptual Analysis

**DOI:** 10.1002/nop2.70747

**Published:** 2026-08-08

**Authors:** Mahmud Ady Yuwanto, Iyus Yosep, Iqbal Pramukti, Ati Surya Mediawati

**Affiliations:** ^1^ Doctoral Study Program in Medical Science, Faculty of Medicine Universitas Padjadjaran Sumedang Indonesia; ^2^ Departemen of Fundamental Nursing, Faculty of Health Sciences Universitas Dr. Soebandi Jember Indonesia; ^3^ Department of Community and Mental Health Nursing, Faculty of Nursing Universitas Padjadjaran Sumedang Indonesia; ^4^ Departemen of Fundamental Nursing, Faculty of Nursing Universitas Padjadjaran Sumedang Indonesia

**Keywords:** caring, clinical judgement, nursing practice, relational accountability, self‐leadership

## Abstract

**Aim:**

This study aims to examine how self‐leadership and caring can be conceptually integrated to strengthen the legitimacy of nursing clinical judgement in complex healthcare environments.

**Design:**

This study adopts a conceptual analysis design.

**Methods:**

A conceptual analysis approach was used to synthesise theoretical perspectives from self‐leadership scholarship and caring theory to clarify the internal conditions that support accountable clinical decision‐making. Self‐leadership was selected because it offers a framework for understanding the self‐regulatory processes underlying intentional professional action, whereas caring theory conceptualises nursing as a moral‐relational practice grounded in human dignity and ethical responsibility. Integrating these theories enabled examination of how regulatory capacity and moral orientation converge to strengthen relational accountability in nursing clinical judgement.

**Results:**

This conceptual analysis proposes a relational accountability framework comprising three interrelated components: regulatory reflexivity, moral‐relational orientation and accountable enactment. Regulatory reflexivity supports intentional reasoning and behavioural alignment, moral‐relational orientation directs decision‐making towards relational responsibility and patient dignity and accountable enactment reflects the transparent communication and justification of clinical decisions within relational care contexts.

**Conclusions:**

Relational accountability offers a theoretical basis for strengthening the legitimacy of nursing clinical judgement by integrating internal regulatory capacity with caring‐based ethical orientation. The framework provides directions for future empirical inquiry into accountable decision‐making in nursing practice.

**Patient or Public Contribution:**

No patient or public contribution.

## 1 Introduction

Clinical judgement is a defining attribute of nursing practice, directly influencing patient outcomes, safety and professional credibility across healthcare settings (Bussard et al. 2024). Foundational scholarship has examined clinical judgement primarily through frameworks of competence, critical thinking and diagnostic accuracy, clarifying how nurses interpret cues, prioritise interventions and evaluate outcomes (Connor et al. 2023; Sezer et al. 2025). These contributions have strengthened understanding of cognitive processes underlying decision‐making. However, contemporary healthcare environments characterised by escalating complexity, rapid technological integration and interprofessional coordination have intensified scrutiny of how nursing judgements are not only accurate, but ethically defensible and publicly accountable (Notarnicola et al. 2025; Zumstein‐Shaha and Grace 2023).

Regulatory standards and professional guidelines delineate scope of practice and expected competencies, yet they do not fully explain what renders a clinical judgement legitimate within everyday relational contexts of care (Mendívil‐Pérez et al. 2025). Emerging evidence suggests that nurses frequently encounter tensions between institutional demands and professional commitments, particularly when performance metrics or system pressures constrain relational engagement (Koivisto et al. 2024; Zhang et al. 2025). These tensions indicate that clinical judgement cannot be reduced to technical proficiency or procedural compliance alone; legitimacy requires more than correct decisions it requires defensible and ethically grounded enactment.

Recent leadership and organisational scholarship further emphasise that professional legitimacy is increasingly evaluated through transparency of reasoning, ethical orientation and relational responsiveness rather than outcomes alone (Iddrisu 2025; James and Priyadarshini 2021). In nursing, accountability extends beyond documentation and compliance to include attentiveness to patient vulnerability, dignity and lived experience (Ghanem Atalla et al. 2025; Molina‐Mula and Gallo‐Estrada 2020). Yet these dimensions regulatory competence and moral‐relational orientation are often analysed separately, limiting theoretical understanding of how they interact within enacted clinical judgement (Ali and Khalifa 2025; Luo and Chan 2023). In this paper, relational accountability refers to the alignment of self‐regulatory processes with caring‐based ethical commitments in nursing clinical judgement. Despite growing recognition of competence, ethics and leadership in nursing practice, limited theoretical attention has been given to how these domains converge to sustain accountable judgement.

Self‐leadership scholarship offers insight into the internal regulatory processes through which individuals intentionally shape cognition, motivation and behaviour under complex conditions (Manz 1986; Neck and Houghton 2006). In healthcare contexts, such regulatory capacity has been associated with adaptive performance and reduced emotional strain (Kadović et al. 2022; Kidanemariam et al. 2026). However, while self‐regulation clarifies how practitioners manage internal processes, it does not specify the normative orientation guiding such regulation (Balasi et al. 2023). Without moral anchoring, regulatory strength may facilitate adaptation to institutional demands without necessarily reinforcing relational accountability (Jønsson and Fasano 2025).

Caring theory positions nursing as a moral practice grounded in relational responsibility, ethical intentionality and respect for human dignity (Ghanbari‐Afra et al. 2022; Watson 2007), and empirical evidence indicates that caring climates and ethically oriented leadership strengthen professional engagement and patient trust (El‐Gazar and Zoromba 2021; Poeira and Nunes 2025). However, caring scholarship has not fully explained how moral commitments are internally stabilised within fast‐paced healthcare settings (Cao et al. 2025; Tyack et al. 2025). While moral orientation indicates the ethical direction of nursing action, it does not clarify how this orientation is sustained through intentional regulatory processes during complex clinical encounters. Consequently, limited theoretical attention has been given to how regulatory processes and moral‐relational orientation jointly produces clinically accountable judgement.

Accordingly, this conceptual analysis advances relational accountability as a foundational condition of legitimate nursing clinical judgement. By integrating self‐leadership as a source of internal regulatory authority with caring as a normative moral orientation, this paper reframes clinical judgement as an enacted practice of accountable agency. Legitimacy is proposed to emerge not merely from cognitive competence or procedural adherence, but from the deliberate alignment of internal regulation with relational responsibility and ethical defensibility in practice. Therefore, the aim of this conceptual analysis is to clarify how self‐leadership and caring can be integrated to strengthen relational accountability in nursing clinical judgement.

## 2 Methods

### 2.1 Design

This study employed a principle‐based conceptual analysis to enhance conceptual clarity and examine the coherence, applicability and boundaries of existing knowledge related to relational accountability in nursing clinical judgement (Penrod and Hupcey 2005). Principle‐based approaches are particularly useful when concepts have been discussed across multiple bodies of literature but remain theoretically fragmented or insufficiently clarified. Given that nursing clinical judgement has often been examined through separate lenses of competence, ethics and leadership, this approach was considered appropriate for exploring how self‐leadership and caring may be integrated to strengthen relational accountability in nursing practice. Rather than seeking to establish definitive attributes of a concept, principle‐based conceptual analysis aims to enhance conceptual clarity by critically examining the coherence, applicability and boundaries of existing knowledge.

### 2.2 Data Sources and Literature Selection

Literature relevant to this conceptual analysis was identified through structured searches of four electronic databases: Scopus, Web of Science, PubMed and CINAHL, supplemented by manual screening of reference lists from seminal theoretical publications and key review articles. Searches were conducted between January and February 2026. No strict publication date restriction was applied because both foundational theoretical works and recent nursing scholarship were considered necessary to preserve conceptual continuity and adequately examine the evolution of the focal concepts.

Search terms were developed iteratively and combined using Boolean operators. The principal search terms included ‘self‐leadership’, ‘caring theory’, ‘human caring’, ‘professional accountability’, ‘ethical competence’, ‘moral agency’, ‘reflective practice’, ‘clinical judgement’ and ‘nursing.’ Additional combinations such as ‘self‐leadership AND nursing’, ‘clinical judgement AND caring’ and ‘accountability AND nursing practice’ were used to identify literature addressing the relationships among the focal concepts.

Eligible sources included peer‐reviewed theoretical papers, conceptual articles, systematic reviews and empirical studies published in English that contributed to understanding self‐leadership, caring, accountability, or nursing clinical judgement. Publications were included if they (a) provided theoretical insight into the focal concepts, (b) examined their application within nursing or healthcare contexts, or (c) informed interpretation of their interrelationships relevant to accountable clinical decision‐making. Seminal theoretical works were retained when necessary to preserve conceptual integrity and trace the historical development of key ideas.

Sources were excluded if they were editorials, commentaries lacking substantive conceptual contribution, conference abstracts without full reports, non‐English publications, or studies unrelated to the theoretical development or practical application of the concepts under investigation. The purpose of these criteria was not to achieve exhaustive coverage but to ensure conceptual relevance and analytical depth consistent with principle‐based conceptual analysis.

The search strategy was adapted to the indexing characteristics of each database while maintaining conceptual consistency across sources. Controlled vocabulary (e.g., MeSH terms in PubMed) was used where available, whereas title, abstract and keyword searches were employed in Scopus, Web of Science and CINAHL. Literature identification and preliminary screening were conducted by the first author, with conceptual relevance, eligibility decisions and analytical interpretation subsequently discussed among all authors until consensus was reached. Rather than aiming for exhaustive retrieval, literature selection prioritised conceptual richness, theoretical relevance and explanatory contribution to the development of the proposed framework.

### 2.3 Analytical Process

The analytical process was guided by the epistemological, pragmatic, linguistic and logical principles of principle‐based concept analysis to critically evaluate the conceptual foundations and interrelationships among self‐leadership, caring and relational accountability within nursing clinical judgement (Penrod and Hupcey 2005). The epistemological principle was applied to examine the maturity and clarity of knowledge surrounding self‐leadership, caring, accountability and clinical judgement within the nursing literature. Pragmatic considerations focused on the usefulness and applicability of these concepts in contemporary nursing practice. Linguistic analysis explored consistency in terminology and conceptual meanings across disciplines, while logical analysis examined the relationships, distinctions and theoretical compatibility among the concepts under investigation.

Through this iterative analytical process, self‐leadership was interpreted as a source of internal regulatory authority, whereas caring was conceptualised as a moral‐relational orientation guiding professional action. The convergence of these dimensions informed the development of relational accountability as a conceptual framework for understanding how nursing clinical judgement becomes professionally justifiable, transparent and responsive within complex healthcare environments.

Specifically, the epistemological principle was used to evaluate the conceptual maturity and theoretical consistency of each construct across the nursing literature. The pragmatic principle examined the usefulness of each concept in explaining contemporary nursing practice. Linguistic analysis compared conceptual definitions and terminology across theoretical traditions to identify conceptual overlap or ambiguity. Finally, logical analysis synthesised relationships among the concepts to determine whether relational accountability represented conceptual redundancy, refinement, or theoretical integration. This iterative comparison ultimately informed the development of the proposed relational accountability framework.

Throughout the analysis, conceptual interpretations were refined through repeated comparison across the four analytical principles rather than through a linear sequence of steps. Interpretive decisions were based on the consistency of conceptual meanings across the literature, their relevance to contemporary nursing practice and their capacity to explain relationships that were insufficiently addressed by existing theories. Discrepancies in interpretation were resolved through iterative discussion among the authors until conceptual coherence was achieved. This process enabled the identification of regulatory reflexivity, moral‐relational orientation and accountable enactment as the principal dimensions of the proposed relational accountability framework.

## 3 Conceptual Foundations of Relational Accountability in Clinical Judgement

### 3.1 Findings From the Principle‐Based Concept Analysis

Application of the four analytical principles revealed that self‐leadership, caring, accountability and clinical judgement are individually well‐established concepts within nursing scholarship, yet they have largely developed as parallel theoretical traditions with limited conceptual integration. Collectively, the epistemological and pragmatic analyses demonstrated that these concepts contribute complementary dimensions to nursing practice. Self‐leadership explains intentional self‐regulation and adaptive professional behaviour, whereas caring provides the ethical and relational orientation that guides professional action. Existing accountability frameworks primarily emphasise responsibility and compliance but offer limited explanation of the internal processes through which accountable clinical judgement is enacted in complex clinical situations.

The linguistic and logical analyses further identified conceptual overlap among accountability, moral agency, ethical competence and reflective practice, while demonstrating that these concepts are complementary rather than redundant. Although related terminology is often used interchangeably, each concept contributes a distinct explanatory perspective. Their theoretical integration therefore supports relational accountability as a higher‐order integrative conceptual framework that explains how intentional self‐regulation and caring‐based moral orientation converge to enable accountable clinical judgement in nursing practice.

The principal findings derived from the application of the four analytical principles are summarised in Table 1, illustrating how each analytical perspective informed the development of the proposed relational accountability framework.

**TABLE 1:** Analytical evidence supporting the development of the Relational Accountability Framework.

Analytical principle	Key analytical findings	Contribution to the relational accountability framework
Epistemological	Self‐leadership, caring, accountability and clinical judgement are conceptually mature but have largely evolved as separate theoretical traditions.	Established the need for an integrative framework capable of explaining how these concepts collectively support legitimate nursing clinical judgement.
Pragmatic	Existing theories explain complementary aspects of nursing practice but do not adequately describe how accountable clinical judgement is enacted in complex care environments.	Identified the practical value of integrating internal self‐regulation with caring‐based ethical orientation to strengthen accountable professional action.
Linguistic	Accountability, moral agency, ethical competence and reflective practice share overlapping terminology despite representing distinct conceptual emphases.	Clarified conceptual boundaries and distinguished relational accountability from related constructs while reducing conceptual ambiguity.
Logical	Self‐leadership and caring are theoretically compatible and mutually reinforcing rather than competing explanations of professional practice.	Supported the conceptualisation of relational accountability as a higher‐order integrative framework explaining the convergence of regulatory reflexivity, moral‐relational orientation and accountable enactment in nursing clinical judgement.

### 3.2 Self‐Leadership as Internal Regulatory Authority

The principle‐based concept analysis identified self‐leadership as the principal explanatory mechanism underlying intentional professional action in nursing clinical judgement. Across the epistemological and pragmatic analyses, self‐leadership consistently emerged as the construct that best explains how nurses regulate cognition, emotion and behaviour to support accountable clinical judgement. In contemporary healthcare environments characterised by rapid clinical change, competing priorities and interprofessional coordination, nurses frequently make decisions under conditions of uncertainty. In such contexts, the legitimacy of clinical judgement depends not only on external standards but also on the nurse's internal capacity to regulate cognition, emotion and behavioural intention in real time. Self‐leadership theory conceptualises individuals as agents who intentionally shape their thinking and actions through structured self‐regulatory strategies (Manz 1986; Neck and Houghton 2006). Within healthcare settings, such regulatory capacity has been associated with adaptive performance and effective professional functioning under demanding conditions (Choi and Kim 2014; Kim et al. 2025).

Applied to clinical judgement, self‐leadership supports alignment between reasoning, intention and action, enabling nurses to articulate and defend their decisions rather than merely execute tasks (Duignan et al. 2024; Jeong and Lee 2023). However, regulatory strength alone does not ensure ethical legitimacy in clinical decision‐making (Goktas and Grzybowski 2025). Without normative orientation, self‐regulation may reinforce efficiency or compliance while remaining morally indeterminate. Consequently, regulatory authority requires moral grounding to ensure that professional agency remains aligned with ethically responsible nursing practice rather than institutional instrumentalism.

### 3.3 Caring as Normative Grounding

The concept analysis further identified caring as the moral‐relational foundation that complements self‐leadership within accountable nursing practice. Across the pragmatic and logical analyses, caring consistently emerged as the ethical orientation that gives normative direction to self‐regulatory processes during clinical judgement. Caring theory conceptualises nursing as a moral‐relational practice grounded in responsibility to human dignity and relational presence (Watson 2007; Wei and Watson 2019). Rather than framing practice as task execution, caring scholarship emphasises ethically intentional engagement with patient vulnerability. Within clinical judgement, caring provides the moral orientation that shapes how nurses interpret patient needs, prioritise interventions and justify decisions in ethically meaningful ways. Empirical evidence further indicates that caring climates and ethically oriented leadership are associated with enhanced professional engagement and patient trust (Fang et al. 2025; F. Zhang et al. 2024).

However, moral commitment alone does not ensure stability in complex healthcare environments. Without deliberate regulatory modulation, the relational demands of caring may intensify emotional strain and vulnerability (Alrashedi et al. 2025; Nabi Foodani et al. 2024). Caring therefore clarifies the ethical direction of clinical judgement but does not fully explain how such orientation is intentionally sustained during complex decision‐making processes. Together, these findings indicate that neither self‐leadership nor caring alone sufficiently explains accountable clinical judgement, thereby providing the conceptual rationale for their integration within the proposed relational accountability framework.

### 3.4 Integration: The Architecture of Relational Accountability

Building on the conceptual gap identified in the Introduction, relational accountability is conceptualised as the condition in which regulatory authority and moral‐relational orientation converge in enacted clinical judgement. It refers to the deliberate alignment of self‐regulatory processes with caring‐based ethical commitments, enabling nurses to exercise judgement that is not only clinically sound but also accountable within relational contexts of care.

Distinct from conventional professional accountability centred on compliance and documentation (Cummings et al. 2018; Mendívil‐Pérez et al. 2025), relational accountability emphasises ethical responsiveness and defensibility in interpersonal clinical encounters. Within this framework, self‐leadership strengthens reflective regulation and intentionality, while caring anchors decision‐making in relational responsibility (Manz 1986; Neck and Houghton 2006; Swanson 1991; Watson 2007). Through their integration, clinical judgement is reframed as accountable professional agency, where the convergence of regulated reasoning and moral orientation sustains both the internal coherence and public defensibility of nursing practice.

To further clarify the contribution of relational accountability, it is important to distinguish this concept from several related constructs frequently discussed in nursing scholarship. Professional accountability has traditionally emphasised responsibility, transparency and adherence to professional and organisational standards (Cummings et al. 2018; Mendívil‐Pérez et al. 2025), whereas ethical competence focuses on the knowledge and skills required to recognise and address ethical issues in practice (Ghanbari‐Afra et al. 2022; Lechasseur et al. 2018). Likewise, moral agency highlights the capacity to act in accordance with ethical values and professional obligations (Bandura 2001; Rushton 2023), while reflective practice promotes self‐awareness and continuous learning through critical examination of experience (Salem et al. 2025; Schön 2009; Younas 2020). Although each of these concepts contributes to understanding professional nursing practice, none explicitly explains how self‐regulatory processes and caring‐based moral commitments converge in enacted clinical judgement. Table 2 summarises these distinctions and highlights the unique contribution of relational accountability as an integrative framework for understanding legitimate nursing clinical judgement.

**TABLE 2:** Distinguishing relational accountability from related concepts in nursing practice.

Concept	Definition and primary focus	Contribution to nursing practice	Distinction from relational accountability
Professional accountability (Cummings et al. 2018; Mendívil‐Pérez et al. 2025)	Refers to the obligation of nurses to adhere to professional standards, legal requirements and organisational policies governing practice.	Promotes responsibility, transparency and compliance with professional expectations.	Relational accountability extends beyond compliance by emphasising the ethical defensibility and relational responsiveness of clinical judgement within patient and interprofessional contexts.
Ethical competence (Ghanbari‐Afra et al. 2022; Lechasseur et al. 2018)	Represents the knowledge, skills and dispositions required to recognise ethical issues and make morally appropriate decisions in practice.	Supports ethical sensitivity and sound ethical reasoning in complex situations.	Relational accountability incorporates ethical orientation but additionally specifies how ethical commitments are enacted through self‐regulatory processes and made visible in practice.
Moral agency (Bandura 2001; Rushton 2023)	Describes the capacity of individuals to act intentionally in accordance with moral values and professional obligations.	Reinforces nurses' responsibility to advocate for patients and uphold ethical principles.	Relational accountability shares a concern for ethical action but further explains the internal regulatory mechanisms through which morally grounded judgements are sustained and communicated in complex environments.
Reflective practice (Salem et al. 2025; Schön 2009; Younas 2020)	Involves the deliberate examination of experiences, assumptions and actions to enhance professional learning and future practice.	Facilitates self‐awareness, learning and improvement in clinical reasoning.	Relational accountability incorporates reflective processes while explicitly integrating caring‐based moral orientation and accountable enactment as conditions of legitimate clinical judgement.
Relational accountability (Present analysis)	Refers to the deliberate alignment of self‐regulatory processes with caring‐based ethical commitments, enabling nurses to enact clinical judgements that are transparent, ethically defensible and responsive to relational contexts of care.	Provides an integrative framework linking regulatory reflexivity, moral‐relational orientation and accountable enactment to support legitimate nursing clinical judgement.	Conceptualises legitimacy as an enacted form of accountable professional agency emerging through the convergence of self‐leadership and caring.

Rather than proposing an entirely new theory of accountability, the findings of the principle‐based concept analysis support the interpretation of relational accountability as a higher‐order integrative conceptual framework that synthesises complementary dimensions of existing nursing scholarship. Professional accountability explains regulatory responsibility, ethical competence emphasises ethical knowledge, moral agency highlights intentional ethical action and reflective practice supports ongoing professional learning. Collectively, the epistemological, pragmatic, linguistic and logical analyses demonstrated that these concepts address distinct yet complementary dimensions of nursing practice rather than representing conceptually redundant perspectives. This analytical synthesis supports their integration into a coherent explanatory framework that clarifies how intentional self‐regulation and caring‐based moral orientation converge during nursing clinical judgement. Accordingly, the framework should be understood as a theoretical refinement and integrative process model that integrates previously separate constructs into a coherent explanatory model for legitimate nursing clinical judgement, rather than replacing existing theories of accountability.

## 4 Relational Accountability Framework for Nursing Clinical Judgement

### 4.1 Judgement Legitimacy as Enacted Accountability

Clinical judgement in nursing has traditionally been evaluated through indicators such as diagnostic accuracy, timeliness and adherence to professional standards (Dehghani 2024; Doyon and Raymond 2025). Although these criteria remain essential, they do not fully capture the conditions under which clinical judgement becomes professionally credible and relationally trustworthy. Contemporary healthcare environments increasingly require transparency of reasoning, ethical responsiveness, and the capacity to justify decisions within interprofessional and patient‐centred contexts (Huesmann et al. 2023; Mendonça 2026).

In this analysis, legitimacy refers to the ethical and professional defensibility of clinical judgement within relational and institutional contexts. Legitimate judgement is therefore not merely procedurally correct but also articulable, ethically grounded and responsive to patient vulnerability. From this perspective, relational accountability reframes legitimacy as enacted accountability, emerging through the visible convergence of regulatory reflexivity and caring‐based moral orientation in clinical action.

### 4.2 Core Components of Relational Accountability

Relational accountability arises from the dynamic integration of three interdependent components that collectively shape the enactment of defensible clinical judgement: regulatory reflexivity, moral‐relational orientation and accountable enactment. Regulatory reflexivity refers to the deliberate regulation of cognition and affects through reflective monitoring during clinical encounters, enabling nurses to critically interpret evolving patient conditions, recognise the influence of emotional and contextual factors and recalibrate reasoning accordingly. Such reflexive regulation strengthens clinical reasoning and enhances the defensibility of professional decisions (Feller et al. 2023; Ren et al. 2026). Complementing this internal regulatory capacity, moral‐relational orientation anchors judgement in attentiveness to dignity, vulnerability and the contextual meaning of patients' experiences. Drawing on caring theory, this orientation frames clinical interpretation as an ethically grounded engagement with patients rather than a purely technical assessment of clinical indicators (Watson 2007; Wei and Watson 2019).

**FIGURE 1:**
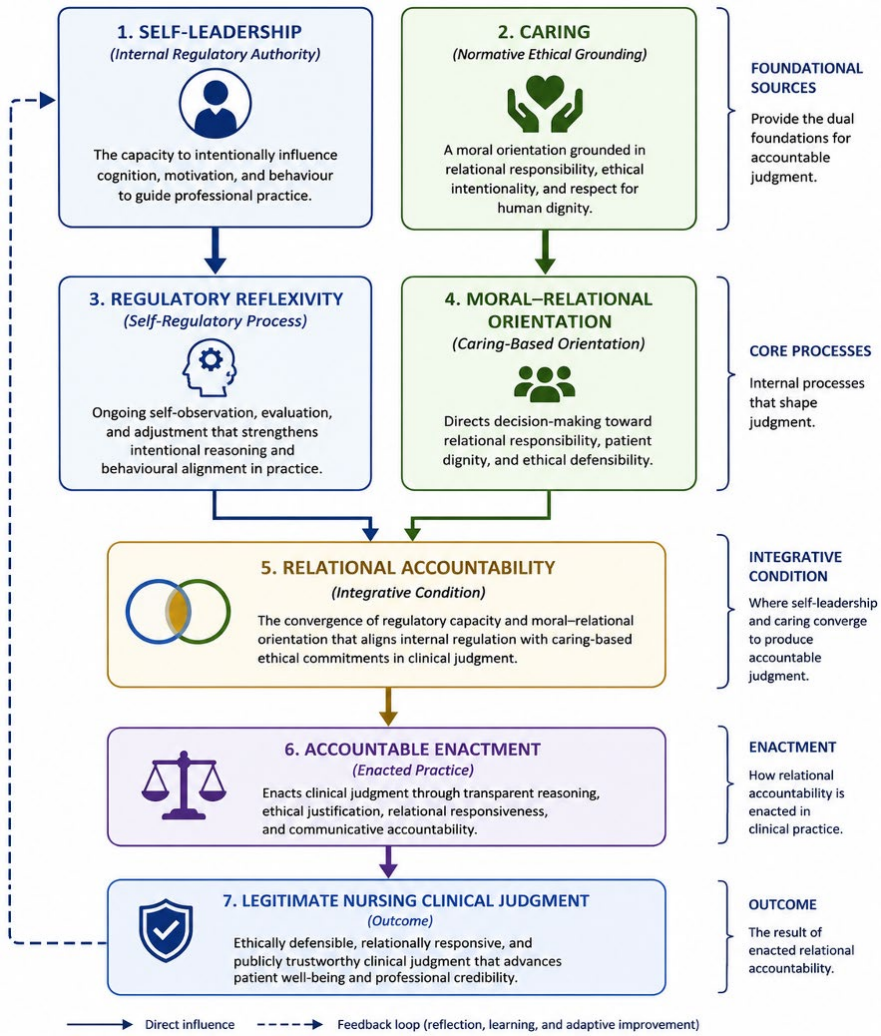
Relational Accountability Framework for Legitimate Nursing Clinical Judgement The framework illustrates how self‐leadership functions as a source of internal regulatory authority through regulatory reflexivity, while caring provides moral‐relational orientation as the normative grounding of nursing practice. Their integration gives rise to relational accountability, which is enacted through explicit justification, ethical justification and relational responsiveness. Through accountable enactment, nursing clinical judgement becomes contextually appropriate, relationally responsive and publicly legitimate.

The third component, accountable enactment, represents the outward expression of clinical judgement through transparent reasoning and ethically responsive action within interprofessional and patient‐facing contexts. It involves articulating the rationale for decisions, engaging in collaborative dialogue and demonstrating responsiveness to relational dynamics in care delivery. Such transparency and communicative defensibility are increasingly recognised as central to professional credibility and safety culture in contemporary healthcare systems (Golitaleb and Safdari 2026; Haskins and Roets 2022). Together, these components illustrate how relational accountability is realised in practice: regulatory reflexivity enables intentional reasoning, moral‐relational orientation directs judgement towards ethically meaningful ends, and accountable enactment renders clinical decision‐making visible, communicable and professionally legitimate within relational and organisational contexts. Figure 1 presents the proposed framework illustrating how self‐leadership and caring converge through relational accountability to support legitimate nursing clinical judgement.

### 4.3 Dynamic Integration and Public Defensibility

Relational accountability is not a static attribute but an emergent property arising from the integration of regulatory reflexivity and caring‐based moral orientation. Regulatory reflexivity stabilises ethical commitment under conditions of clinical complexity, while caring provides the normative direction that guides regulatory processes towards ethically appropriate action. Through this convergence, clinical judgement is enacted as accountable professional agency rather than discretionary authority.

This perspective extends beyond competence‐based models and emotional intelligence frameworks by clarifying how internal regulation and ethical commitment jointly constitute legitimacy in practice. In contemporary healthcare systems, where professional decisions are subject to increasing scrutiny, such integration is critical for sustaining interprofessional credibility and public trust (Lawongsa 2025; Rushton 2023). By situating legitimacy within enacted relational accountability, the framework shifts analytic attention from isolated cognitive accuracy towards the architecture of defensible professional agency in complex care environments.

### 4.4 Observable Indicators of Relational Accountability

Although relational accountability is conceptual in orientation, its enactment can be inferred through observable clinical behaviours. These include the transparent articulation of clinical reasoning in interprofessional dialogue, explicit ethical justification of decisions in patient communication, responsiveness to patient narratives and the readiness to recalibrate judgements when new contextual information emerges. Such practices reflect evidence linking open communication, reflective reasoning and collaborative engagement with strengthened professional credibility and patient safety (Cunha et al. 2025; Lee et al. 2024).

Within complex team‐based care environments, transparent justification of clinical decisions has become central to professional trustworthiness and safety culture (Berkhout et al. 2025; Mistri et al. 2023). Similarly, responsiveness to patient narratives reflects caring‐based relational engagement associated with greater patient trust and perceived quality of care (Curcio et al. 2024; Ewunetu et al. 2025). These observable practices illustrate how relational accountability becomes visible in everyday nursing practice and provide a basis for future empirical research examining the defensibility, legitimacy and accountability of nursing clinical judgement.

## 5 Implications for Clinical Practice, Education and Leadership

### 5.1 Clinical Practice

Strengthening the legitimacy of nursing clinical judgement requires more than technical competence. Nurses must also develop the capacity to regulate their reasoning processes while sustaining ethical and relational responsiveness towards patients. The Relational Accountability Framework emphasises the alignment of internal regulatory processes with caring‐based moral commitments, positioning clinical judgement as an intentional practice grounded in both reflective regulation and relational responsibility.

In clinical settings, strategies such as reflective clinical rounds, structured ethical case discussions and interdisciplinary dialogue may support this alignment by encouraging nurses to articulate the reasoning and ethical considerations underlying their decisions (Hwu and Pai 2025; Safavi et al. 2022). Reframing accountability not merely as procedural compliance but as the integration of professional self‐regulation and relational responsibility may strengthen the ethical defensibility and professional credibility of nursing clinical judgement in complex healthcare environments.

### 5.2 Nursing Education

Educational preparation for nursing clinical judgement should extend beyond models that emphasise analytic accuracy alone. Integrating self‐regulatory training such as reflective calibration and cognitive reframing with structured engagement in caring theory may better equip students to enact relationally accountable judgement in complex clinical environments (Akdeniz and Duygulu 2025; Ramluggun and Morning 2025). This integration supports the development of both reflective regulation and ethical‐relational awareness in clinical decision‐making.

Educational strategies including simulation‐based learning, reflective journaling and guided ethical dialogue can further strengthen these capacities by encouraging students to examine the reasoning and moral considerations underlying their decisions. Embedding explicit discussions of legitimacy and ethical defensibility within simulation and case‐based learning may enhance students' ability to articulate, justify and enact clinical judgements that are both professionally accountable and relationally responsive.

### 5.3 Leadership and Governance

Leadership approaches that foster psychological safety and encourage ethical dialogue are essential for enabling relational accountability in nursing practice (Hessler et al. 2025; Mrayyan and Askar 2025). When leaders promote openness, reflective communication and mutual respect, nurses are better able to articulate the clinical reasoning and ethical considerations underlying their decisions. Organisational cultures that support open dialogue and reflective learning further strengthen these conditions by encouraging reasoned decision‐making and collaborative judgement in complex clinical situations.

At the governance level, systems that prioritise transparency and learning‐oriented accountability rather than punitive oversight may reinforce the legitimacy of nursing clinical judgement. Policies that emphasise shared responsibility, reflective evaluation and constructive feedback help cultivate professional integrity and trust within healthcare teams. In this perspective, organisational trustworthiness is closely linked to the relational accountability enacted by nurses at the point of care, where ethically grounded and transparently reasoned decisions sustain both professional credibility and public confidence in nursing practice.

## 6 Limitations and Future Research

This conceptual analysis is theoretical in scope and does not provide empirical validation of the proposed framework. The integration of self‐leadership and caring is interpretive and may require contextual refinement across diverse healthcare systems and cultural settings. Additionally, emphasising internal regulatory capacity risks underestimating structural determinants that shape professional enactment. Relational accountability should therefore be understood not as an individualistic solution to systemic strain but as one dimension within a broader ecology of organisational, relational and policy influences. Empirical testing is necessary to evaluate its explanatory and predictive validity within varied clinical contexts.

Although the proposed framework is theoretical, it provides a foundation for future empirical investigation. Future research should examine whether self‐leadership contributes to regulatory reflexivity during nursing clinical judgement, whether caring orientation strengthens relational accountability, and whether relational accountability explains accountable clinical judgement beyond existing concepts such as professional accountability and ethical competence. Empirical studies involving instrument development, structural equation modelling, longitudinal designs and implementation research are needed to evaluate the construct validity, explanatory value and practical applicability of the proposed framework across diverse nursing contexts.

## 7 Conclusion

Clinical judgement in nursing requires more than technical competence and procedural adherence. As healthcare environments grow increasingly complex and publicly scrutinised, legitimacy depends on the integration of internal regulatory authority and caring‐based moral orientation. Fragmented approaches that treat competence, ethics and leadership as parallel domains leave unresolved how judgement becomes relationally defensible in practice.

By advancing relational accountability as the enacted convergence of self‐leadership and caring, this analysis clarifies the internal conditions through which clinical judgement becomes ethically grounded, transparent and publicly defensible. The proposed framework provides a theoretical basis for future empirical investigation into how relational accountability may contribute to professional credibility, interprofessional trust and legitimate nursing clinical judgement.

## Author Contributions

**Iyus Yosep:** data curation, supervision, resources, methodology. **Iqbal Pramukti:** methodology, data curation, supervision, resources. **Mahmud Ady Yuwanto:** investigation, funding acquisition, writing – original draft, methodology, validation, writing – review and editing, software, formal analysis, project administration, data curation, resources. **Ati Surya Mediawati:** data curation, supervision, resources, methodology.

## Funding

The publication of this article was supported by Universitas Padjadjaran through the International Reputable Journal Publication Funding Programme under Grant No. 1719/UN6.RKT/Kep/HK/2025. The first author also received a scholarship from the Indonesian Ministry of Higher Education, Science and Technology under Grant No. 3927/UN6.RKT/HK.07.00/2025 through the Beasiswa Pendidikan Indonesia (BPI) programme, Ministry of Higher Education, Science and Technology (Grant No. 015646/PPAPT.1.2/BPI.06/02/2025).

## Ethics Statement

As this study was based exclusively on the conceptual analysis and synthesis of published literature, it did not involve human participants, patient data, or identifiable personal information. Consequently, ethics approval and informed consent were not required.

## Conflicts of Interest

The authors declare no conflicts of interest.

## Data Availability

Data sharing not applicable to this article as no datasets were generated or analysed during the current study.
